# Aiming for agency and authenticity in assessment

**DOI:** 10.1007/s40037-018-0484-z

**Published:** 2018-11-08

**Authors:** Christopher Harrison

**Affiliations:** Brooklands Medical Practice, Manchester, UK

Assessment designers have a lot to consider. As well as the traditional psychometric principles of validity and reliability, an assessment’s educational impact is, appropriately, now receiving increasing attention. Unfortunately, the impact is often not positive. Although the need to ensure ‘assessment for learning’ is frequently mentioned, in practice the summative assessment culture is often so dominant and powerful that it acts as a barrier to learners processing feedback in any meaningful way [1].

In an interesting paper in this issue of the journal, Ricci and colleagues [2] argue persuasively that we can promote the goal of ‘assessment for learning’ by engaging students as active stakeholders in assessment. When student opinions on assessment have been sought in the past, the focus has typically been on simplistic notions of acceptability while interpretations of validity have been left to psychometricians. However, Ricci argues that it is crucial to recognize the agency of learners, which is their capacity to act and make choices within the constraints of their context. When we do, we find that they are insightful into how assessments will impact on future learning. For example, the students in Ricci’s study could recognize whether an assessment was an authentic reflection of real-world practice.

These findings resonate with other research into the factors in an assessment culture which influence learners’ receptivity to feedback [3]. Learners’ agency was promoted by providing elements of choice within assessments as they gave students an opportunity to demonstrate (to themselves and their assessors) how successfully they had mastered their acquisition of knowledge. Agency was also promoted by feedback systems which allowed learners to act with some degree of autonomy. Promoting agency appeared to foster learners’ aspirations towards excellence. However, assessment cultures frequently act in ways which inhibit agency. High-stakes assessments can stifle learning by acting as hurdles to negotiate, especially if they do not feel sufficiently authentic and relevant to future learning in the clinical workplace [3]. A worrying finding from this study was the revelation that feedback given on the wards was often ignored as it was felt to harm the chances of passing the summative assessment [3]. This may explain why learners often choose to prepare for clinical skills assessments away from the workplace [4]. These factors are not just relevant to undergraduate education, they also influence clinicians’ acceptance of physician validation systems [5].

Enabling students to be active agents in assessment will not, of course, solve all our assessment problems. When students were given an opportunity to be active stakeholders in redesigning an assessment program, they recognized the problem with lack of authenticity in current assessment practices and the resulting inhibitory effect on clinical workplace learning [6]. However, they were equally worried about the potential loss of standardization and perceived assessment fairness if authenticity was promoted. The summative assessment paradigm remains dominant with most assessment stakeholders, including students; changing this culture remains a challenge [6].

Ricci believes that students will actively interpret their assessment scores. However, provision of feedback is no guarantee that it will be used in a meaningful way [7]. A child receiving a nicely-wrapped present instinctively knows what to do; a learner presented with the gift of feedback does not automatically have the same skills. Mentoring of a learner from a trusted adviser can help interpretation of feedback and in so doing can promote agency [3].

Unfortunately, many assessment programs pay no more than lip service to agency and authenticity. Instead, assessment cultures frequently seem deeply rooted in the behaviourist approach to education; students are punished for failing and rewarded for passing assessments. This is perhaps surprising as medical learning institutions have increasingly adopted more active approaches to learning. This misalignment between assessment and learning strategies can have negative consequences. A curriculum can have many strategies to encourage active learning, but if the assessment programs are not designed with care, learners will unsurprisingly adopt superficial approaches to learning [8].

As a clinician, I am reminded of the huge changes in clinical practice over the last few decades. In the internet era, the notion of a patient as a passive recipient of healthcare is thankfully becoming increasingly obsolete. Instead, patients are, or should be, much more active partners in the clinical consultation [9]. Incorporating the patient perspective is not just courteous, it helps doctors to make more correct diagnoses and promotes efficiency in the consultation [10]. Similarly, the concept of a student passively accepting the rewards and punishments of an assessment program seems increasingly archaic and difficult to justify.

If these proposed changes to the assessment culture continue to seem too radical, perhaps it would be helpful to reflect that the need for change has been recognized for more than 100 years. Sir William Osler, the famous physician, clearly understood the adverse impact the summative assessment culture had on medical education:

*We make the examination the end of education, not an accessory in its acquisition. The student is given the impression that he is in the school to pass certain examinations and I am afraid the society in which he moves grinds this impression into the soul *[11].

Among his suggested remedies were elements of what we would now call an assessment for learning culture. He recognized the need for authentic assessment by advocating frequent observations and judgements in the workplace. He had even considered the need to foster students’ agency, quoting a former colleague approvingly:

*Let us emancipate the student and give him the time and opportunity for the cultivation of his mind, so that in his pupillage he shall not be a puppet in the hands of others, but rather a self-relying and reflecting being* [11].

Changing an assessment culture will always be challenging, but there are potential rewards for teachers, students and, most importantly, patients, who have much to gain from doctors who are open to receiving, interpreting and acting on feedback. As a starting point, recognizing the agency of the learner and thinking carefully about the authenticity of our assessments seems a good approach.
